# Design of Carbon Dots Photoluminescence through Organo-Functional Silane Grafting for Solid-State Emitting Devices

**DOI:** 10.1038/s41598-017-05540-5

**Published:** 2017-07-14

**Authors:** Kazumasa Suzuki, Luca Malfatti, Masahide Takahashi, Davide Carboni, Fabrizio Messina, Yasuaki Tokudome, Masanori Takemoto, Plinio Innocenzi

**Affiliations:** 1 Laboratorio di Scienza dei Materiali e Nanotecnologie, D.A.D.U., Università di Sassari, CR INSTM, Palazzo Pou Salit, Piazza Duomo 6, 07041 Alghero (Sassari), Italy; 2 Department of Materials Science, Graduate School of Engineering, Osaka Prefecture University, Sakai, Osaka 599-8531 Japan; 3 International Institute for Nano/Meso Materials Science, Osaka Prefecture University, Sakai, Osaka 599-8531 Japan; 4 Dipartimento di Fisica e Chimica, Università degli Studi di Palermo, Via Archirafi, 36 90123 Palermo Italy

## Abstract

Advanced optical applications of fluorescent carbon dots (C-dots) require highly integrated host-guest solid-state materials with a careful design of C-dots – matrix interface to control the optical response. We have developed a new synthesis based on the grafting of an organo-functional silane (3-glycidyloxypropyltrimethoxysilane, GPTMS) on amino-functionalized C-dots, which enables the fabrication of highly fluorescent organosilica-based hybrid organic-inorganic films through sol-gel process. The GPTMS grafting onto C-dots has been achieved via an epoxy–amine reaction under controlled conditions. Besides providing an efficient strategy to embed C-dots into a hybrid solid-state material, the modification of C-dots surface by GPTMS allows tuning their photoluminescence properties and gives rise to an additional, intense emission around 490 nm. Photoluminescence spectra reveal an interaction between C-dots surface and the polymeric chains which are locally formed by GPTMS polymerization. The present method is a step forward to the development of a surface modification technology aimed at controlling C-dots host-guest systems at the nanoscale.

## Introduction

Carbon dots (C-dots) are a unique class of fluorescent nano-objects with resistance to photo-bleaching, low toxicity, excellent biocompatibility and good water solubility^1–4^. Fluorescent C-dots with high quantum efficiency and controlled emission wavelength are increasingly becoming an alternative to semiconductor quantum dots (SQDs) such as CdSe and ZnS. A high quantum yield (more than 40%) is however necessary to challenge SQDs performances. This goal is generally achieved by doping the C-dots with nitrogen^5–8^, although the interactions between nitrogen atoms and carbon structures have not been yet elucidated. It is, in fact, difficult to make a quantitative comparison among different types of C-dots, because their properties, such as the size distribution, the surface functionalization, the carbon core structures (graphitic or amorphous) and the number of defects, are strongly dependent on the synthesis.

C-dots fluorescence is, in general, attributed to two contributions: the emission from the carbon-core and surface states^9^. The carbon-core is usually characterized by a sp^2^ carbon structure similar to graphite, potentially affected by quantum confinement effects due to the nanosize, whereas surface electronic transitions are related to functional groups^10–12^. The preparation of nitrogen-doped C-dots has been achieved through a variety of different molecules, such as ethylenediamine^5, 13^, urea^14, 15^, and chitosan^6, 16^. Although there is a strong empirical evidence of the nitrogen atoms contribution on C-dots fluorescence, the chemical-physical mechanisms which are behind the fluorescence have not yet been fully understood. When C-dots structure is composed by sp^2^ hybridized carbon atoms, graphitic-nitrogen can be accommodated in both core and surface sites. On the other hand, pyridinic, pyrrolic and amino-N atoms are located only at the surface sites. A theoretical study using density functional theory (DFT) by Sarkar *et al*.^17^ has suggested that the C-dots properties depend on the location of nitrogen: graphitic-N centers induce a red-shift of absorption, while other surface-located nitrogen atoms do not cause a red-shifting. However, other experiments suggest that also surface nitrogen atoms have an influence on the transition wavelengths, despite only a few systematic studies have been published so far. For instance, Kwon *et al*. have succeeded in fine-tuning C-dots PL and have obtained green, orange and red emissions^18^. To achieve this goal, post-functionalization of aniline derivatives onto graphene quantum dots, a sub-class of C-dots, has been used. Ding *et al*. reported that C-dots containing abundant nitrogen atoms (pyridinic-N, amino-N and pyrrolic-N), can be separated by their polarity using column chromatography^19^. A full-color range emission of C-dots in visible region with a high quantum yield has been obtained by this method. In the same publication, the authors have supposed that the PL tuning can be caused by the surface defective states, showing a different oxidation state. These findings suggest that C-dots fluorescence can be tuned by controlling the state of the surface nitrogen atoms in C-dots structure. The electron affinity of nitrogen atoms can be modified through a grafting reaction with an organic functional group. On the other hand, the surface functionalization plays also a fundamental role for embedding C-dots into solid matrices. This is a technological requirement that should be carefully considered for developing innovative optical devices. Indeed, despite a decade of intense research^20–22^, examples of solid-state applications of C-dots are still not yet common. Understanding the chemical reactions between C-dots and an inorganic host is a mandatory step for the chemical design of nanomaterials as it enables to cross the boundaries between nanocomposites and hybrid materials.

In this work, we have therefore modified the C-dots surface using a molecule which is able to play a double role of a surface modifier through a grafting reaction and network former of the host matrix. 3-glycidyloxypropyltrimethoxysilane (GPTMS), an organically modified alkoxide bearing an epoxide, has been selected for this purpose^23, 24^. The epoxides, in particular, can react with primary and secondary amines by a nucleophilic attack which enables grafting through epoxy-opening^25^. At the same time the Si(OCH_3_)_3_ groups in GPTMS participate to the formation of the silica network. The choice of GPTMS also provides other indirect advantages: firstly, the GPTMS aliphatic chain is expected to prevent the C-dots aggregation, which is responsible for fluorescence quenching, especially at high concentrations. Quenching is particularly detrimental for optoelectronic devices and sensors which require solid-state emitting materials and should be carefully avoided^26^. Secondly, the use of GPTMS is expected to improve the stability of the C-dots in solid-state. Hybrid organic-inorganic GPTMS matrices show a higher transparency and photo-stability with respect to pure organic materials^27^. GPTMS-grafted C-dots are therefore expected to be a class of more stable phosphors to be dispersed in solid-state. Overall, we show that this route allows fabricating stable organosilica-based hybrid films wherein the fluorescent properties of C-dots are preserved or even enhanced in comparison to the liquid phase.

## Results and Discussion

The functionalization of C-dots with GPTMS has been designed to allow a full integration into a hybrid organic-inorganic material through covalent bonding and to modulate the photophysical properties. We have, therefore, selected and optimized the synthesis to control the C-dots structure and PL properties.

### C-dots structure and properties

To synthesize the C-dots with graphitic and well defined structure, we have used a hydrothermal reaction of citric acid (CA) and ethylene diamine (ED)^5^,﻿﻿ following an optimized procedure described in detail in the methods section. ED has been used as the N-source because it does not form dimers and oligomers leading to a reproducible synthesis. The presence of amino-groups on the C-dots available for the epoxy-amine grafting has been verified by FTIR spectroscopy. The spectrum (Figure S1 in Supplementary Information) shows that the functional groups on the C-dots are mainly secondary amines which are predominant with respect to primary amines.

**Figure 1:**
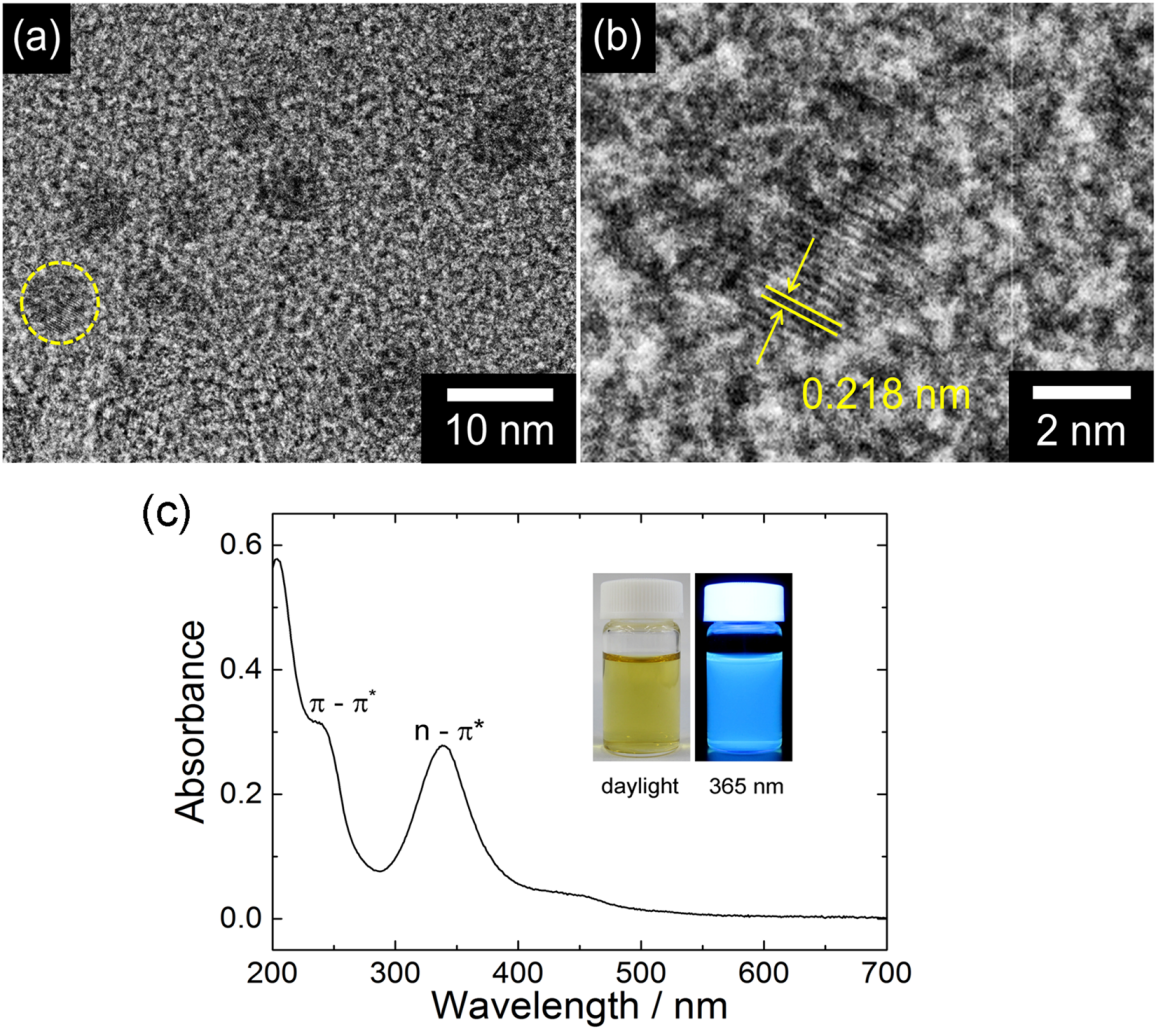
(**a**) TEM image of the C-dots. (**b**) TEM image of the yellow dotted line in Fig. 1a. The lattice fringes correspond to (110) planes of graphite. (**c**) UV-Vis absorption spectrum of C-dots in water. The inset shows the picture of the C-dots dispersion in water under daylight and upon UV (365 nm) irradiation.

Although the C-dots are partially composed by an amorphous structure containing vinyl C=C and C-C bonds, the graphitic structure is clearly observed by TEM. The C-dots show lattice planes with a spacing of 0.218 nm, which corresponds to (110) in-plane lattice periodicity of graphite (Fig. 1a and b)^28^. The C-dots appear as spherical particles with an average size of 8 nm in accordance with previous findings^5^.

Figure 1c shows the UV-Vis absorption spectrum of C-dots in water in the range 200–700 nm. The absorption band peaking at 240 nm is due to the π-π* transitions of aromatic C=C bonds. We attribute the band at 338 nm to n-π* transitions of C=O^29^. This band is also featured by a weak and broad wing extending well above 400 nm, as observed in many other C-dot systems^17, 29^. The C-dots in water show an intense blue photoluminescence upon illumination with UV-light (365 nm), as shown in the inset of Fig. 1c.

**Figure 2:**
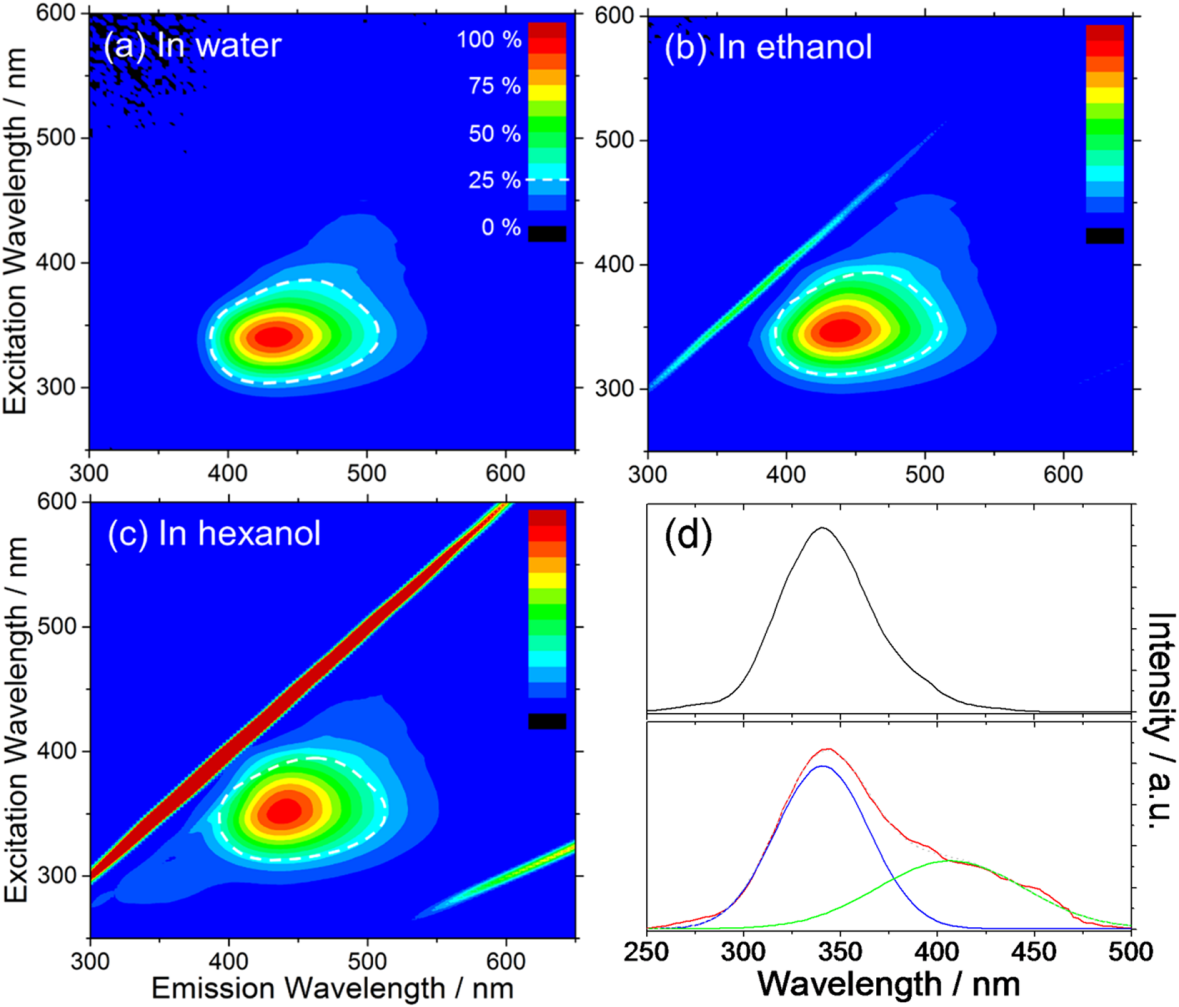
Photoluminescence excitation-emission-intensity spectra of C-dots dispersed in different solvents: (**a**) water, (**b**) ethanol, (**c**) hexanol. The intensity is shown in a false color scale. The spectrum region with an emission intensity higher than 25% of C-dots in water is shown as reference (white dotted line). (**d**) Excitation spectra of C-dots in water corresponding to the emission at 430 nm (black line, top) and at 490 nm (red line, bottom). The latter curve is formed by two overlapped components (blue and green lines) that have been separated by deconvolution.

The photoluminescence of C-dots in different solvents has been characterized by recording excitation-emission-intensity spectra (Fig. 2). The overall emission is only slightly modified by changing the solvent, and it is very similar in water, ethanol and hexanol. The weak solvatochromism of the main emission band is also supporting the hypothesis that most of N atoms are located on the C-dots surface. Formation of carbon nitride core structures is, in fact, generally associated to a strong solvatochromism^30^. The shape of the signal shown in Fig. 2 highlights that the emission is mostly excited by the 338 nm n → π* absorption, which is related to C=O surface groups.

Figure 2d shows the excitation spectra of the C-dots collected at 430 nm (top, black line) and 490 nm (bottom, red line), respectively. The excitation curve corresponding to the emission at 490 nm is formed by two overlapped components that we have separated by deconvolution (blue and green lines). The excitation bands peaking at 340 nm (blue band) and 406 nm (green band) are associated to the absorptions at 338 nm and the wing extending above the 400 nm in Fig. 1c, respectively.

### GPTMS grafting onto amino-functionalized C-dots

**Figure 3:**
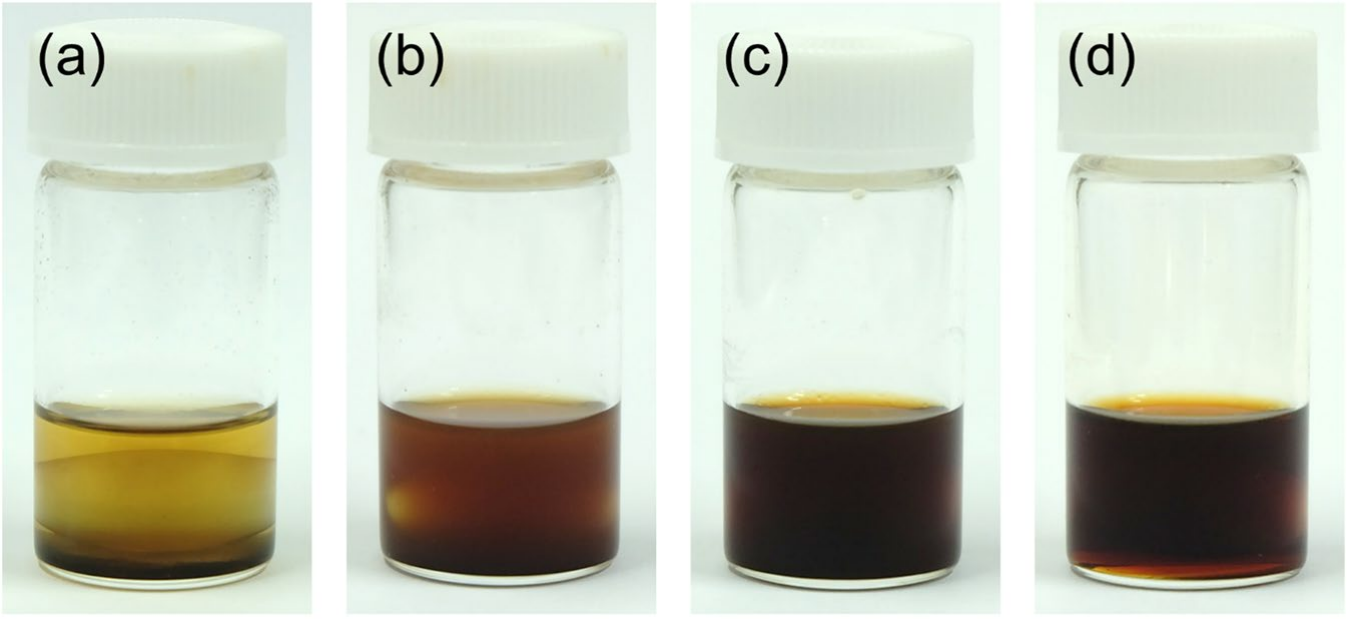
Images of GPTMS – C-dots mixtures at increasing reaction times: (**a**) 1 hour, (**b**) 2 days, (**c**) 5 days and (**d**) 2 weeks.

GPTMS grafting process onto the amino-functionalized C-dots has been monitored by evaluating the C-dots dispersibility in solution and it has been characterized by FTIR and ^1^H-NMR spectra. The different appearance of the GPTMS – C-dots mixture as a function of the reaction time is shown in Fig. 3. When the grafting reaction has not yet occurred, the C-dots are poorly dispersed in the acetone, GPTMS and TiCl_4_ solution. Black precipitates of C-dots are observed at the bottom of the vial after ultrasonication and 1 hour of reaction (Fig. 3a, the yellowish color is mainly due to TiCl_4_ in acetone). As reaction time elapsed, the mixture became cloudy and brownish (Fig. 3a–c); after 2 weeks of reaction, the mixture finally turned into a clear brown sol with no precipitates. This change has been considered as an indication of well-dispersed C-dots into the sol (Fig. 3d). These changes can be used to evaluate the extent of the grafting reaction, which takes 2 weeks to go to completion under the present experimental conditions. After this period, the sol remains stable for at least 3 months in a capped vial, suggesting that condensation of GPTMS does not take place. This result shows that GPTMS-grafting is more favored with respect to condensation in a non-aqueous system with aprotic solvents.

**Figure 4:**
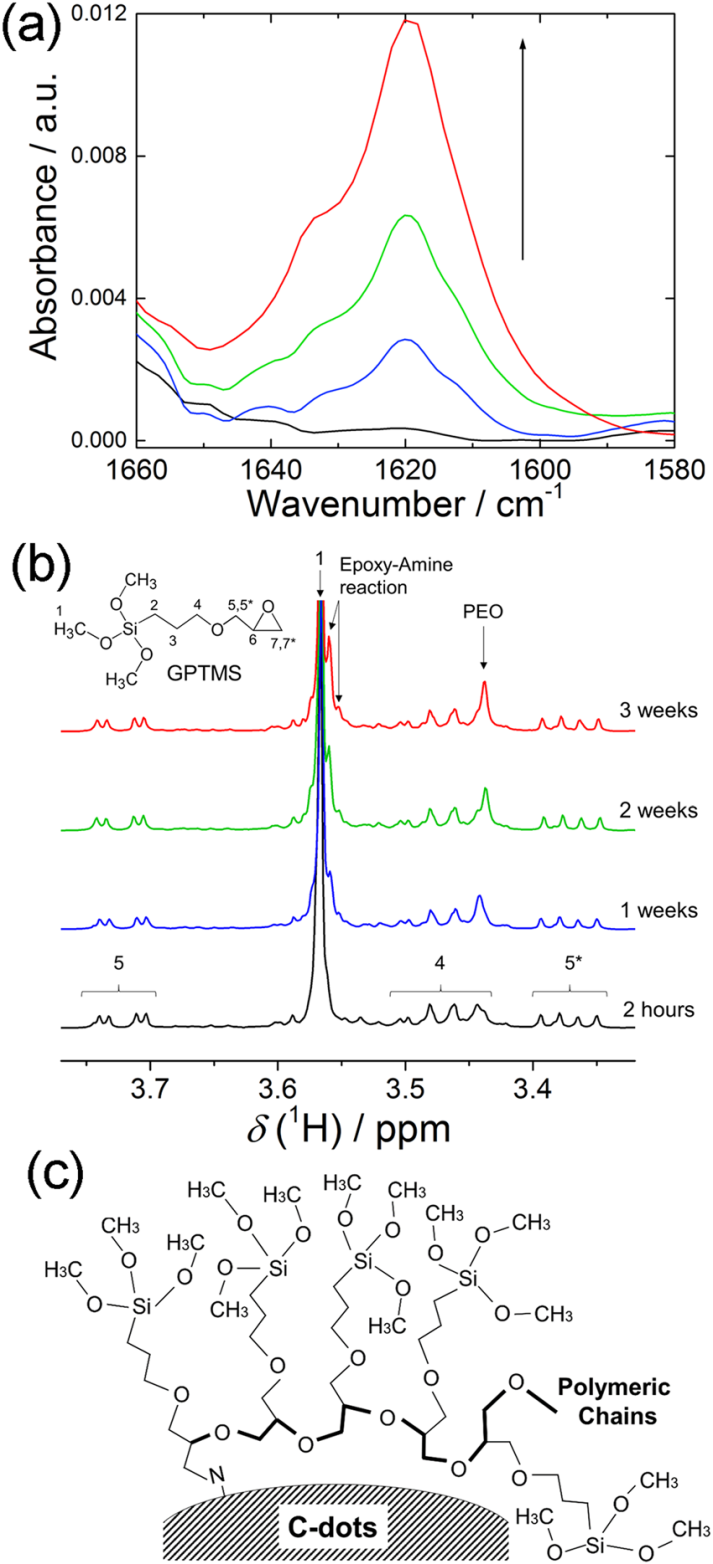
(**a**) FTIR absorption spectra in the range 1660–1580 cm^−1^ of the GPTMS – C-dots grafting sol at different reaction times: 1 hour (black line), 2 days (blue line), 5 days (green line) and 2 weeks (red line). The arrow is a guide for eyes to indicate the increase of the vinyl bands with the reaction time. (**b**) ^1^H-NMR spectra of GPTMS – C-dots grafting sol with reaction times up to 3 weeks. (**c**) Formation of ethylene oxide chains with grafting through epoxy-ring opening and the polymerization between GPTMS molecules at the C-dots surface.

To confirm the grafting process, FTIR spectroscopy has been performed at different reaction times (1 hour, 2 days, 5 days and 2 weeks) as shown in Fig. 4a. Since the C-dots powder is not completely dispersed up to 2 weeks of reaction (Fig. 3), the supernatant sol has been directly dropped onto the diamond window used for ATR measurements. The bands at 1620 and 1640 cm^−1^ which are assigned to the vinyl groups (C=C), increase with time^31^, suggesting an increase of C-dots concentration in the sol (Fig. 4a). This effect has been attributed to a higher C-dots dispersibility induced by the progressive GPTMS grafting onto C-dots; the hydrophobic alkoxy groups in GPTMS, in fact, make the C-dots more soluble in acetone.

^1^H-NMR spectroscopy has been also performed at the different reaction times of the mixture (2 hours, 1 week, 2 weeks and 3 weeks) (Fig. 4b). Besides the presence of GPTMS and acetone (Figure S2), we have observed a noticeable increase of the signals at *δ* = 3.55 and 3.56 ppm with the increase of reaction time. This has been attributed to the grafting reaction of the epoxide rings in GPTMS with the amines on the surface of C-dots^32^. Interestingly, also the peak at 3.44 ppm shows a similar increase with time as the epoxy–amine reaction proceeds. This rising signal can be assigned to the formation of polyethylene oxide (PEO) chains produced by the epoxy-ring opening and the polymerization among the epoxy groups of GPTMS^33^. The PEO chains are obtained by polycondensation of C-OH groups resulted from the epoxy-ring opening. Considering the reaction between amino-groups and epoxide as highly favored, the formation of some PEO chains is likely initiated by a GPTMS molecule chemically bonded to the C-dots surface, giving rise to polymers bearing alkoxysilane side arms (Fig. 4c).

### Preparation of the photoluminescent hybrid material

GPTMS-grafted C-dots (GC-dots) and C-dots have been used to prepare films through one-pot sol-gel reaction of an organo-functional silane mixed with the C-dots.

**Figure 5:**
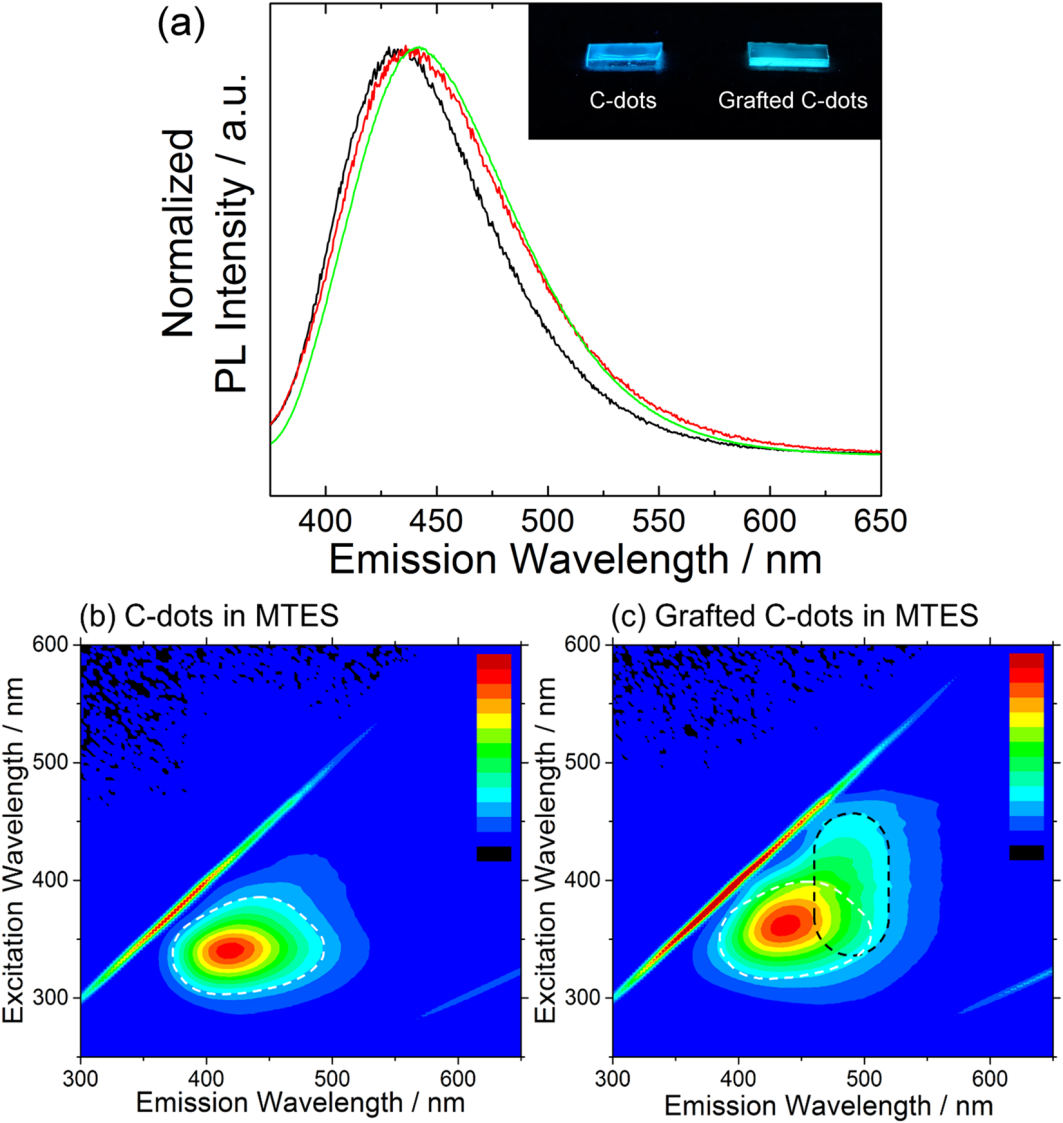
(**a**) Photoluminescence spectra of MTES – C-dots (black line) and MTES – GC-dots (red line) nanocomposite films excited at 365 nm. The emission of C-dots in water (green line) excited at the same wavelength is reported as reference. (**b**,**c**) Excitation-emission-intensity spectra of the films, prepared from pristine C-dots and GPTMS-grafted C-dots, dispersed in a MTES sol. Normalized photoluminescence intensity is reported in false color scale from blue to red (0–100%). The inset of Fig. 5a shows the appearance of the films under UV lamp irradiation (*λ* = 365 nm). The spectrum region with an emission intensity higher than 25% of C-dots in water is shown as reference (white dotted line). The black dotted line represents the estimated new emission area peaked around 490 nm.

Two organosilane precursors, methyltriethoxysilane (MTES) and GPTMS, have been selected to prepare the hybrid materials. The hybrid films prepared from these precursors have a flat surface and a typical thickness of 3.8 μm (MTES) and 6.7 μm (GPTMS), (Figure S3). The GPTMS-grafted C-dots can be easily dispersed in both MTES and GPTMS sols, while an additional ultrasonication process is required for the pristine C-dots to be dispersed in both sols because of the poor dispersibility. By this procedure, described in detail in the methods section, we have successfully obtained highly photoluminescent sol-gel films embedding C-dots. A picture of a typical film under UV illumination is shown in the inset of Fig. 5a.

Figure 5 shows the PL spectra of the MTES films prepared with GC-dots and C-dots. When exciting by a 365 nm light which is close to the main n → π* absorption in Fig. 1c, a 5 nm red-shifted emission (from 433 nm to 438 nm) is observed in the MTES **–** GC-dots in comparison to the MTES – C-dots samples (Fig. 5a), and both are very similar to C-dots in water. However, excitation-emission-intensity mapping provides more detailed information on the luminescence characteristics, and reveals the differences between the two samples.

In fact, as shown in Fig. 5b, the emission area of the MTES – C-dots sample is similar to that of the original C-dots dispersed in water, yielding a strong, single emission associated with the absorption peak at 338 nm. In contrast, the spectrum of the MTES **–** GC-dots shows a second emission area around 490 nm (Fig. 5c, black dotted line), which is very much enhanced with respect to Fig. 2a. The map indicates that the additional 490 nm emission has the highest intensity upon excitation around 400 nm, which is at lower energy with respect to the main absorption band.

**Figure 6:**
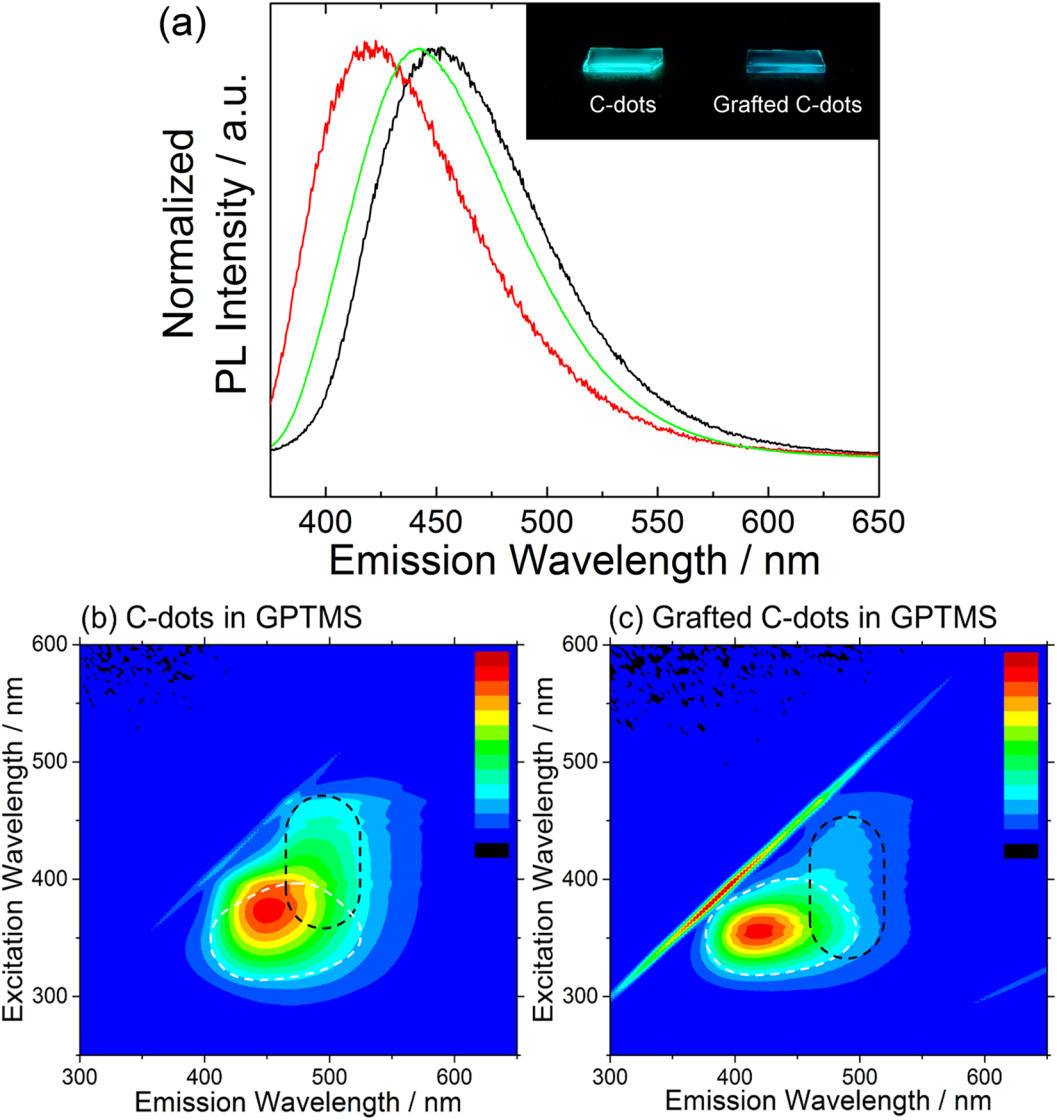
(**a**) Photoluminescence spectra of GPTMS – C-dots (black line) and GPTMS – GC-dots (red line) nanocomposite films excited at 365 nm. The emission of C-dots in water (green line) excited at the same wavelength is reported as reference. (**b**,**c**) excitation-emission-intensity spectra of the films, prepared from pristine C-dots and GPTMS-grafted C-dots, dispersed in GPTMS sol through sol-gel process. Photoluminescence intensity, normalized by the peak-top count, is reported in false color scale from blue to red (0–100%). The inset of Fig. 6a shows the appearance of these films under UV lamp irradiation (*λ* = 365 nm). The spectrum region with an emission intensity higher than 25% of C-dots in water is shown as reference (white dotted line). The black dotted line represents the estimated new emission area peaked at around 490 nm.

Figure 6 shows the PL spectra of the GPTMS – GC-dots and GPTMS – C-dots systems. In contrast to the MTES matrix, when excited at 365 nm considerable shifts of the emission are observed with respect to C-dots in water, towards the blue or red for GPTMS – GC-dots and GPTMS – C-dots, respectively (Fig. 6a). The inset of the Fig. 6a shows the appearance of the films under UV lamp irradiation (*λ* = 365 nm): the blue emission color of the GPTMS – GC-dots film, is clearly different from the cyan emission of the GPTMS – C-dots film. The spectra in Fig. 6b and c reveal that both samples display the additional emission around 490 nm excited around 400 nm, which is independent of the grafting as it was observed for the MTES samples as well (Fig. 5c). Furthermore, the relative PL intensity of the emission band around 490 nm with respect to the original C-dots emission in the GPTMS – GC-dots is smaller than in GPTMS – C-dots. This is an opposite response with respect to the MTES matrix.

**Figure 7:**
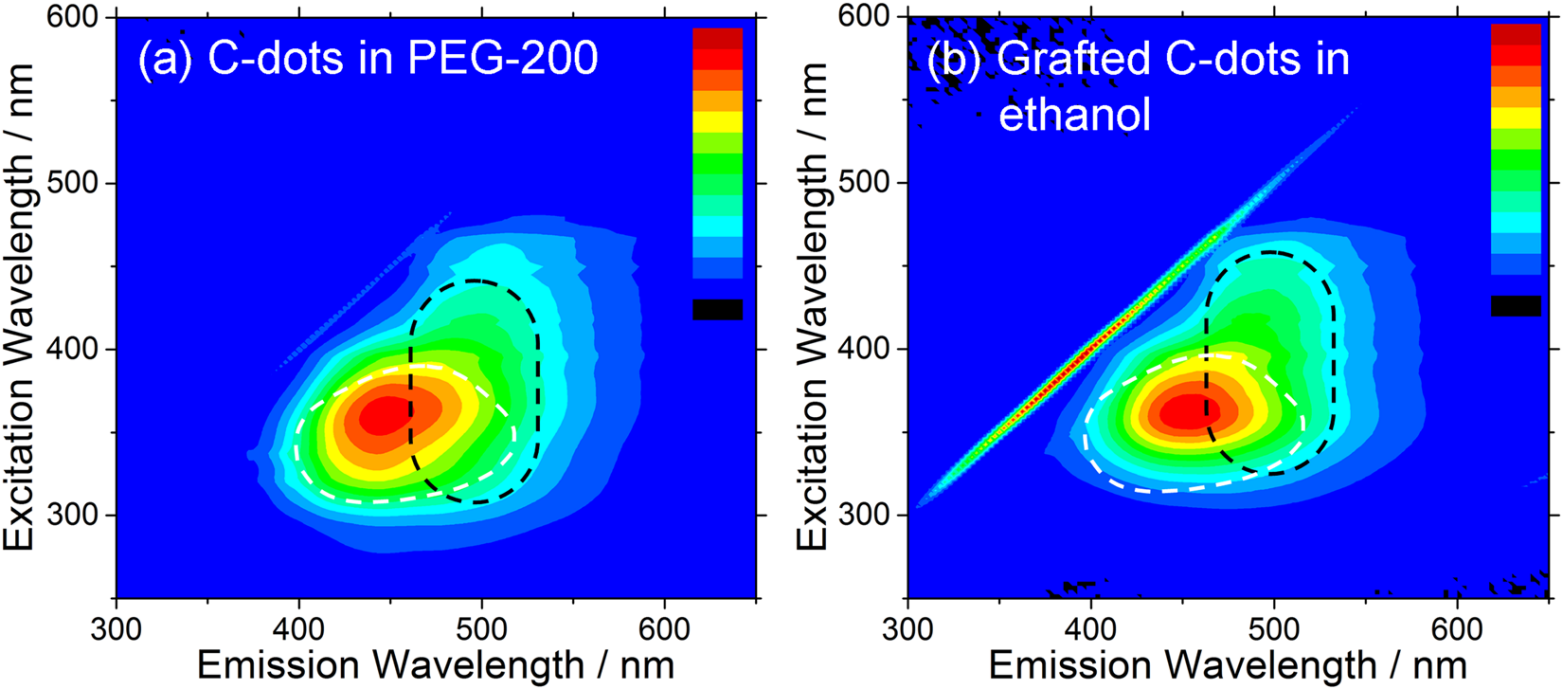
Photoluminescence excitation-emission-intensity spectra of C-dots dispersed in (**a**) polyethylene glycol 200 (PEG-200) and (**b**) grafted C-dots dispersed in ethanol. The spectrum region with an emission intensity higher than 25% of C-dots in water is shown as reference (white dotted line).

Overall, the results of Figs 5 and 6 show that both the position of the main emission around 420–450 nm, and the relative intensity of the additional emission around 490 nm can be tailored, to some extent, by choosing the preparation method of the hybrid material. The photoluminescence quantum yield (PLQY) of the shows films is in the range between 9 and 14%, as shown in Figure S4. From a comparison between Figs 5 and 6, we suppose that the formation of polyethylene oxide (PEO) and diols due to GPTMS functionalization affects the photoluminescence properties by changing the chemical environment around C-dots^33^. To understand how the presence of these polymeric chains changes the C-dots luminescence, PL mapping spectra of the C-dots dispersed in polyethylene glycol 200 (PEG-200) have been measured as reference (Fig. 7a). Indeed, also when dispersed in PEG-200 the C-dots show the new emission component around 490 nm as shown by the black dotted lines. Since the PEG-200 itself does not emit at that wavelength, the origin of the emission band has been attributed to the interaction with the polyethylene oxide chains regardless the presence of GPTMS^34^.

Interestingly a similar PL response is observed for the GC-dots when dispersed in ethanol (Fig. 7b), suggesting that the 490 nm emission somehow involves surface traps localized on amino groups, related to the grafting reaction.

According to previous findings^35^, the C-dots surface interactions with the polymeric molecules, is accountable for the luminescence (peaked at ~490 nm in the present case). We assume that the lower-energy surface traps for a radiative emission is provided by hydrogen bonding interaction with PEG-200, restoring fluorescence at longer wavelengths which is almost absent in other solvents. A similar effect to the surface interaction of C-dots with PEG-200 is induced by grafting, whereby amino-related surface traps are stabilized through the grafting reaction.

These results indicate that the long-wavelength emission of the GC-dots in MTES matrix and observed also in ethanol, is caused by the interaction of polymeric chains on C-dots surface through the grafting reaction. On the other hand, the case of GPTMS-grafted C-dots in GPTMS matrix, where we also observe shifts in the main emission band around 420 nm, requires a more sophisticated explanation. The difference between the two systems, GPTMS – GC-dots and GPTMS – C-dots, in fact, can be only explained on the base of a different chemical environment close to the C-dots surface.

When excited at 365 nm, the GPTMS – GC-dots system shows a different emission apparently shifted towards the blue with respect to the GPTMS – C-dots. This counterintuitive result can be explained by considering the relative intensity of the emission at 490 nm with respect to the GPTMS functionalization. The grafting of epoxides with the amino groups in fact allows creating several “anchoring points” on the C-dots surface that act as spacers, inhibiting a close interaction between the PEO chains and the C-dots surface because of steric hindrance. On the other hand, the C-dots added in one-pot to the GPTMS sol directly interact with the organosilane molecules during sol-to-gel transition. This favors the physical interaction of the C-dots with the PEO chains with respect to chemical grafting. Since the grafted molecules limit the development of polymeric chains near the C-dots surface, the interaction with additional PEO chains is not favored in GPTMS – GC-dots films. On the contrary, in the GPTMS – C-dots system, there is no limitation for polymeric chains to be located near C-dots. Since the amount of GPTMS in the sol-gel reaction is in large excess and the chemical condition for the reaction are different with respect to those of grafting reaction, the strength of PEO interaction in GPTMS – C-dots film is higher (Fig. 6a) causing the PL red shift compared to GPTMS – GC-dots film.

The blue-shifted emission of the GPTMS – GC-dots film can be also explained by the predominant grafting effect with respect to surface interaction. The nucleophilicity of the amines decreases with GPTMS grafting due to the steric effect. This decreases the C-dots polarity causing the blue-shifted emission^19, 36^.

**Figure 8:**
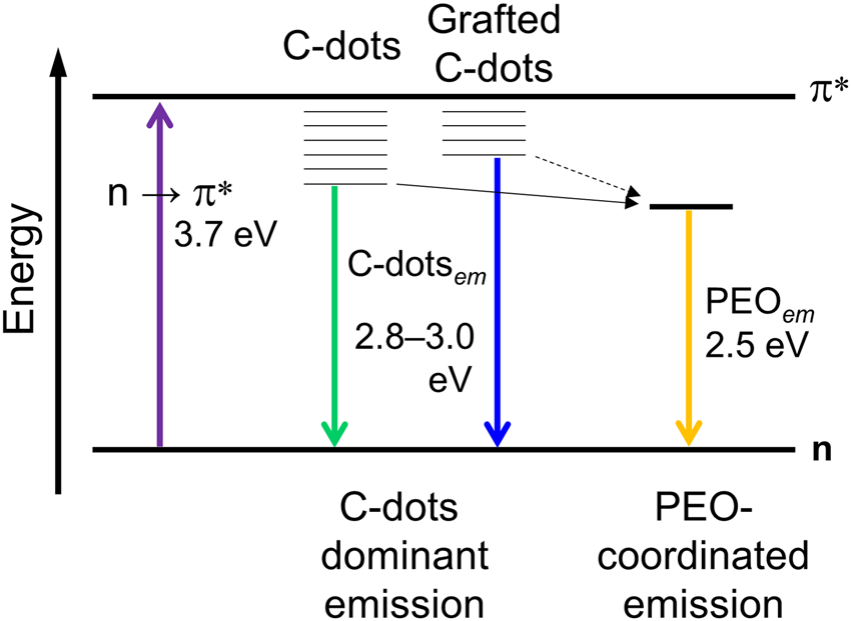
PL energy levels diagram of the GPTMS – GC-dots system.

Figure 8 resumes the electronic transitions involved in the PL of the GPTMS – GC-dots system. At lower energy, 3.7 eV (~338 nm), the absorption attributed to n → π* electronic transition, in surface C=O defects of graphitic C-dots, is observed. The radiative process of the excited electrons mainly occurs in a range of 2.8–3.0 eV (~420–450 nm). Finally, at lowest energy range, we observe the emission around 2.5 eV (~490 nm), whose intensity is strongly enhanced by the PEO interactions. This transition has been correlated to traps energy levels on the C-dots surface due to the amino groups. The intensity changes of the emission at 2.5 eV, which are caused by the interaction with the PEO chains, are the main cause of the overall emission red shifting.

Even if MTES and GPTMS films have different chemical-physical properties^37^, it is reasonable to hypothesize that in both cases the self-polymerization of organosilane can induce the enhanced emission around 490 nm. These experiments clearly point out how the surface affects the emission properties of a system containing fluorescent C-dots. The incorporation of C-dots into an organosilica matrix, in fact, cannot be considered as the simple merging of two different materials, because the interplay in the host-guest system is responsible for remarkable different PL properties depending on the organosilica matrix.

## Conclusion

Hybrid organic-inorganic thin films with C-dots, whose fluorescence is controlled by surface functionalization, have been successfully prepared. A full integration of the host-guest system has been obtained through grafting of the surface amines onto C-dots to silicon alkoxides bearing epoxy groups. The surface functionalization allows a high C-dots loading in the films ensuring a high homogeneity of the host-guest systems. The C-dots functionalization also increases the intensity of the emission band at 490 nm which overlaps to the main emission band at 430 nm. The rise of this new emission band has been attributed to the hydrogen bonding interactions with the polyethylene oxide species formed upon ring opening reactions of 3-glycidoxypropyltrimethoxysilane. The chemical surface modification via molecular grafting induces, therefore, a peculiar change of photoluminescence. In turn, C-dots alone exhibit a different response which is highly sensitive to changes in the host matrix. The combination of the GPTMS-grafted C-dots with sol-gel organosilica appears as a valuable method for preparing solid-state emitting materials, to be used in flexible displays and lasers.

## Methods

### Preparation of amino-functionalized C-dots

Amino-functionalized C-dots were prepared from citric acid and ethylenediamine via hydrothermal synthesis based on an established way^5^. Citric acid monohydrate (1.0 g, 4.76 × 10^−3^ mol) and ethylenediamine (318 μl, 4.76 × 10^−3^ mol) was dissolved in water (10 ml). Then the solution was transferred into a Teflon-lined autoclave (20 ml) and heated at 200 °C for 5 hours with 10 °C/min heating rate from 25 °C. After the hydrothermal treatment, the autoclave was cooled down up to 25 °C without any control of the cooling rate. The product was a brown solution that underwent dialysis in water (2000 MWCO) for 3 days. The final solution was dried at 60 °C to obtain the C-dots powder.

### Organosilica – C-dots Nanocomposite Films Preparation

Organosilica – C-dots nanocomposite films has been prepared by a 2-step process: 1) grafting of GPTMS onto C-dots via epoxy-amine reaction, and 2) films preparation via sol-gel process mixed with the grafting sol of C-dots.

### Grafting of GPTMS onto C-dots

The solution for grafting of GPTMS onto C-dots was prepared under non-aqueous condition. 1 ml of GPTMS, 1 ml of acetone and 5 mg of the amino-functionalized C-dots were mixed together. Then 49 μl of titanium chloride (TiCl_4_) was added as a Lewis acid catalyst for epoxy-ring opening. The molar ratio of GPTMS:TiCl_4_ was 10:1. The mixture was treated with ultrasonication for 10 min, and then the mixture was left to react under stirring for 2 weeks for promoting the epoxy – amine reaction in a closed vial. After 2 weeks of reaction, the mixture changed into a homogeneous brown suspension.

### Films Preparation via sol-gel process

Two types of organically modified silicon alkoxides, methyltriethoxysilane (MTES) and 3-(glycidoxypropyl)trimethoxysilane (GPTMS), were chosen as precursors for the preparation of organic – inorganic hybrid films, MTES – C-dots or GPTMS – C-dots. The MTES and GPTMS precursor sols were prepared using a water/alkoxide = 1:3 (2.70 ml of MTES or 3.75 ml of GPTMS was mixed with 900 μl of H_2_O). The sols were left to react under stirring for 2 days to promote the hydrolysis of the organosilanes and to increase the viscosity of the sol through condensation. 450 μl of the GPTMS-grafted C-dots (GC-dots) solution was finally added to the MTES and GPTMS precursor sols. As reference, MTES and GPTMS hybrid films using pristine C-dots were also prepared. 1.5 mg of the C-dots powder, corresponding to the amount of C-dots in the sol prepared with the grafted C-dots, was used for the preparation of the reference samples. Since the C-dots in powder are difficult to be dispersed into the sol, ultrasonication was performed for 1 hour. The resulting sols were deposited on silica substrate by spin-coating with 500 rpm spinning rate. After spin-coating, the samples were dried at 60 °C for 2 days to promote the condensation of siloxane chains.

### Characterizations

#### Fourier transform infrared (FTIR) spectroscopy

FTIR spectroscopy has been performed to identify the chemical bonding for C-dots grafting sol and the original C-dots powder using an interferometer Bruker infrared Vertex 70 v. The spectra of grafting sol have been recorded in attenuated total reflectance (ATR) mode in a range between 4000 and 400 cm^−1^ by averaging 64 scans with 4 cm^−1^ of resolution. The C-dots powder have been dispersed in a potassium bromide and the spectra have been recorded in transmission mode using the potassium bromide (KBr) pellet in the same range by averaging 256 scans with 4 cm^−1^ of resolution. The background has been evaluated by measuring the signal of KBr. The baseline has been fitted by a concave rubber band correction with OPUS 7.0 software.

#### Nuclear magnetic resonance (^1^H-NMR) spectroscopy

Liquid ^1^H-NMR spectroscopic measurement has been carried out for grafting mixture on a JEOL JNM-ECS400 instrument, operating at a proton frequency equal to 400 MHz. All NMR samples measured have been comprised of CDCl_3_ (containing 1 vol% of TMS) solvent (75 vol%) and the grafting mixture (25 vol%). The NMR tube containing a sample (diameter = 5 mm) has been sealed and kept closed during the full duration of the experiment. The chemical shifts are referenced to TMS (*δ* = 0 ppm).

#### Photoluminescence (PL) spectroscopy

PL spectra of organosilica – C-dots nanocomposite films have been recorded with a “NanoLog” Horiba Jobin Yvon spectrofluorometer, Japan: Excitation-fixed spectra at 365 nm and Excitation-Emission-Intensity PL spectra have been recorded with a 450 W xenon lamp as the excitation source. PL spectra have been collected with an excitation range of 250–600 nm and an emission range of 300–650 nm. PL map spectra of C-dots dispersed in different solvents have been recorded using a quartz cuvette with the NanoLog under the same conditions as films.

#### Photoluminescence quantum yield (PLQY) measurement

PLQY measurement has been performed using the quanta-*φ* (HORIBA) integrating sphere accessory, attached to the “NanoLog” spectrofluorometer. Silica substrate is used as a blank and the coating films deposited on the silica substrate are used as a sample for PLQY measurement. The size of the silica substrate is fixed as 12 mm × 12 mm, and the film coating has been performed by the spin-coating with the rotating rate of 500 rpm for each sample, and the films have been obtained after dried at 60 °C for 2 days. The PLQY value has been determined as average of the three measurements.

#### Surface morphology and thickness measurements

Surface roughness and thickness of the films have been measured using Microfigure Measuring Instrument SURFCORDER ET200 (Kosaka Laboratory Ltd., JAPAN). A part of the film on glass substrate was peeled off by knife, and the microfigure measurement was performed in between substrate and film. The thickness was determined by the average of the thickness of six points in a film. The resolution of the tip is less than 1 nm.

## Electronic supplementary material

Supplementary information
