# Supplementary material for: Design of Carbon Dots Photoluminescence through Organo-Functional Silane Grafting for Solid-State Emitting Devices
Source: Sci Rep. 2017 Jul 14;7:5469. doi: 10.1038/s41598-017-05540-5 (PMC5511139; doi:10.1038/s41598-017-05540-5)
Supplement: Supplementary file 1 — Supplementary information [file 41598_2017_5540_MOESM1_ESM.docx]

**Supporting Information**

**Design of Carbon Dots Photoluminescence through Organo‑Functional Silane Grafting for Solid-State Emitting Devices**

Kazumasa Suzuki,^1,2^ Luca Malfatti,^1,3^ Masahide Takahashi,^2,3^ Davide Carboni,^1,3^ Fabrizio Messina,^4^ Yasuaki Tokudome,^2,3^ Masanori Takemoto,^2^ Plinio Innocenzi.^1,3*^

^1^ Laboratorio di Scienza dei Materiali e Nanotecnologie, D.A.D.U., Università di Sassari, CR INSTM, Palazzo Pou Salit, Piazza Duomo 6, 07041 Alghero (Sassari), Italy.

^2^ Department of Materials Science, Graduate School of Engineering, Osaka Prefecture University, Sakai, Osaka 599-8531, Japan.

^3^ International Institute for Nano/Meso Materials Science, Osaka Prefecture University, Sakai, Osaka 599-8531, Japan.

^4^ Dipartimento di Fisica e Chimica, Università degli Studi di Palermo Via Archirafi, 36 90123 Palermo, Italy.

^*^ email: plinio@uniss.it


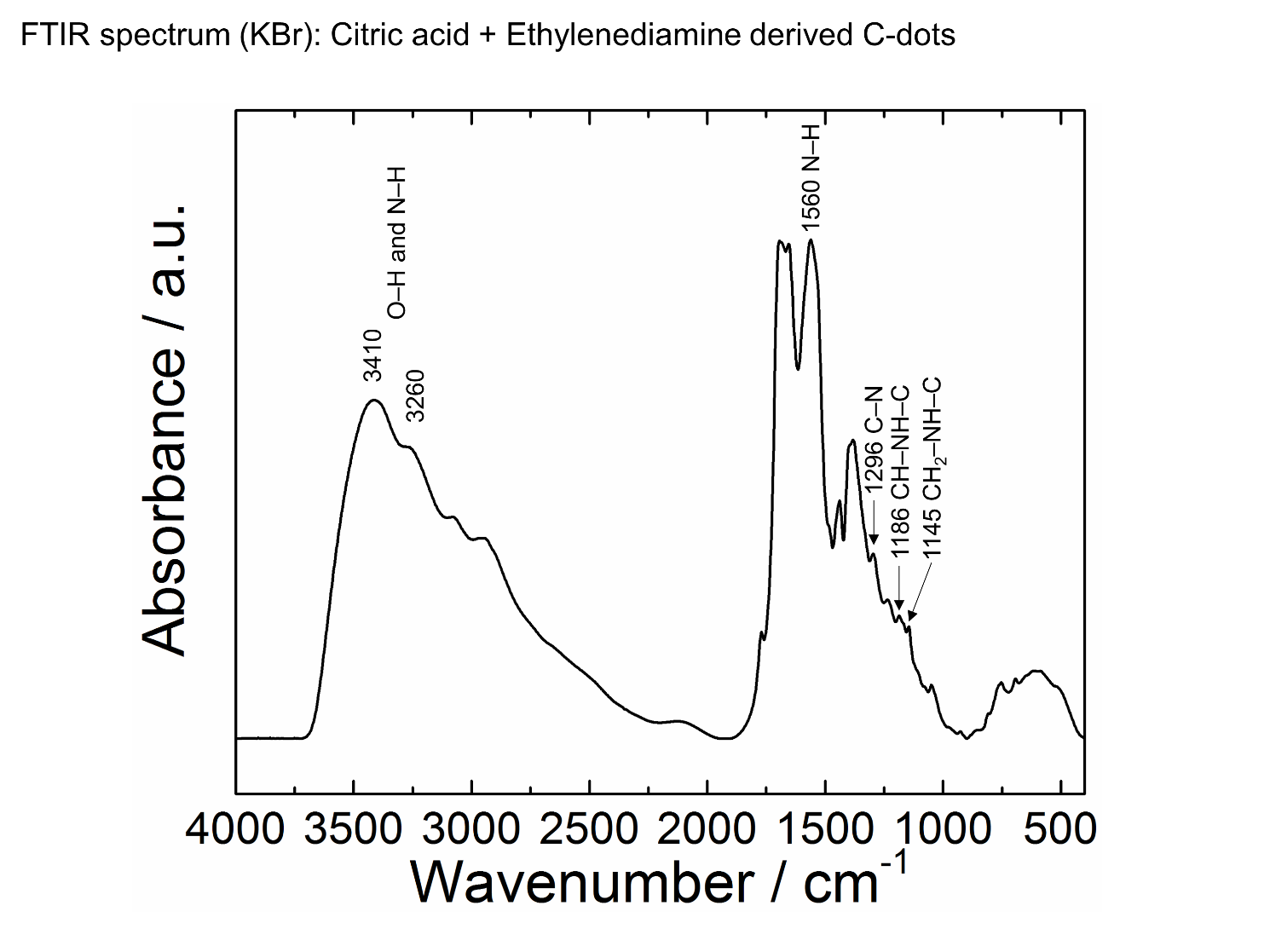


**Figure S1.** FTIR spectrum in the 4000-400 cm^-1^ range of the original C-dots powder in a KBr pellet. The attribution of the significant absorption bands for grafting is described in the figure. Several characteristic bands related to the amines are observed. The bands at 3410 and 3260 cm^-1^ are both assigned to overlapped stretching vibrations between O-H and N-H. The band at 1560 cm^-1^ is attributed to the bending vibration of N‑H, and those at 1296, 1186 and 1145 cm^-1^ are assigned to C-N in aromatic, non‑cyclic secondary amine (CH‑NH‑C) and aliphatic secondary amine (CH_2_-NH-C), respectively. Vibrations attributed to other functional groups such as C=N (1655 cm^-1^), C=O (1695 cm^-1^), C-O (1383 cm^-1^), C=C (3075 cm^-1^), C-H (2950 cm^-1^) and O-H (broad absorption around 3300-3500 cm^-1^) are also observed.


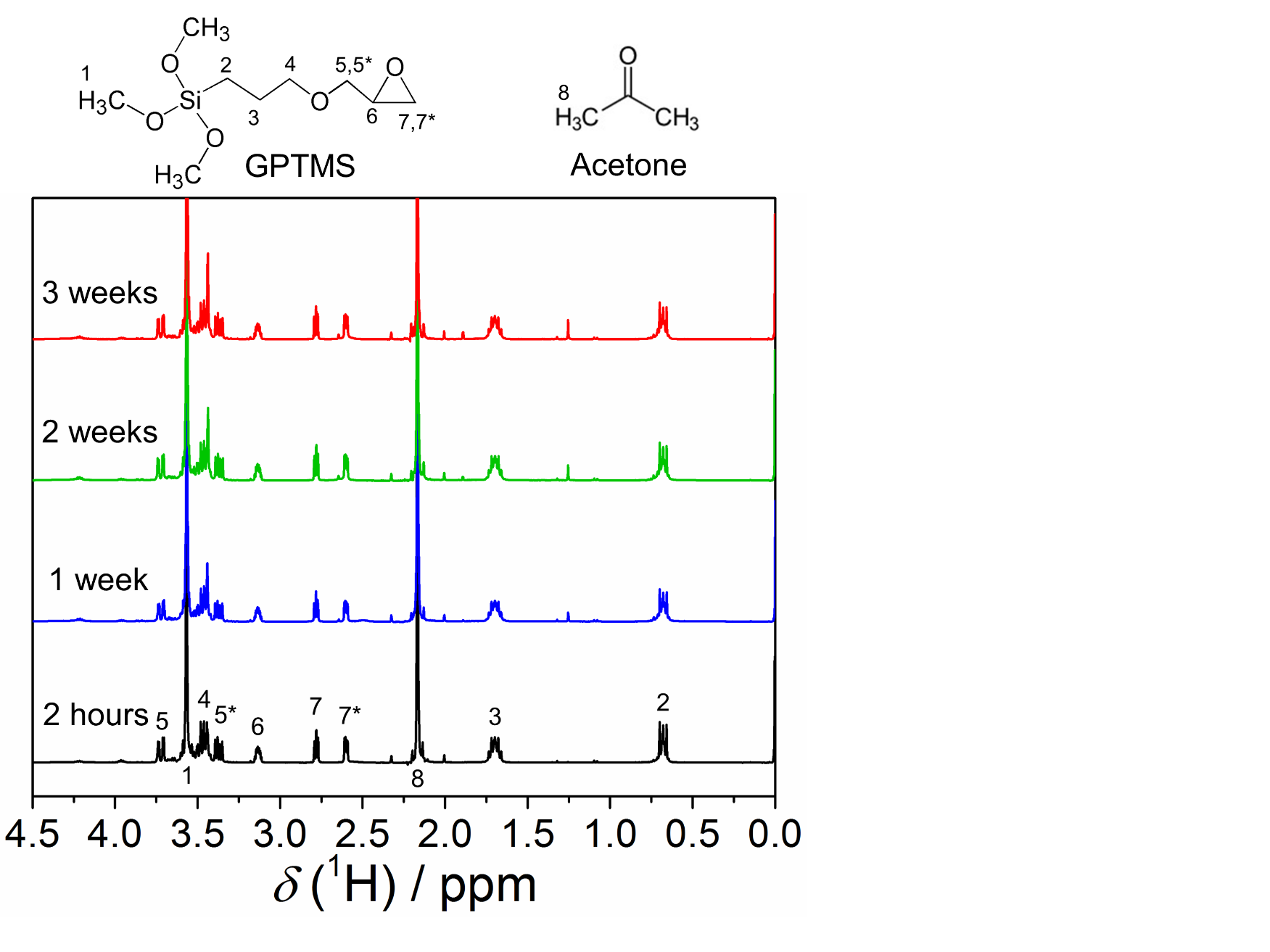


**Figure S2.** ^1^H-NMR spectra of GPTMS – C-dots grafting sol with different reaction time up to 3 weeks and peak assignment of GPTMS and acetone.


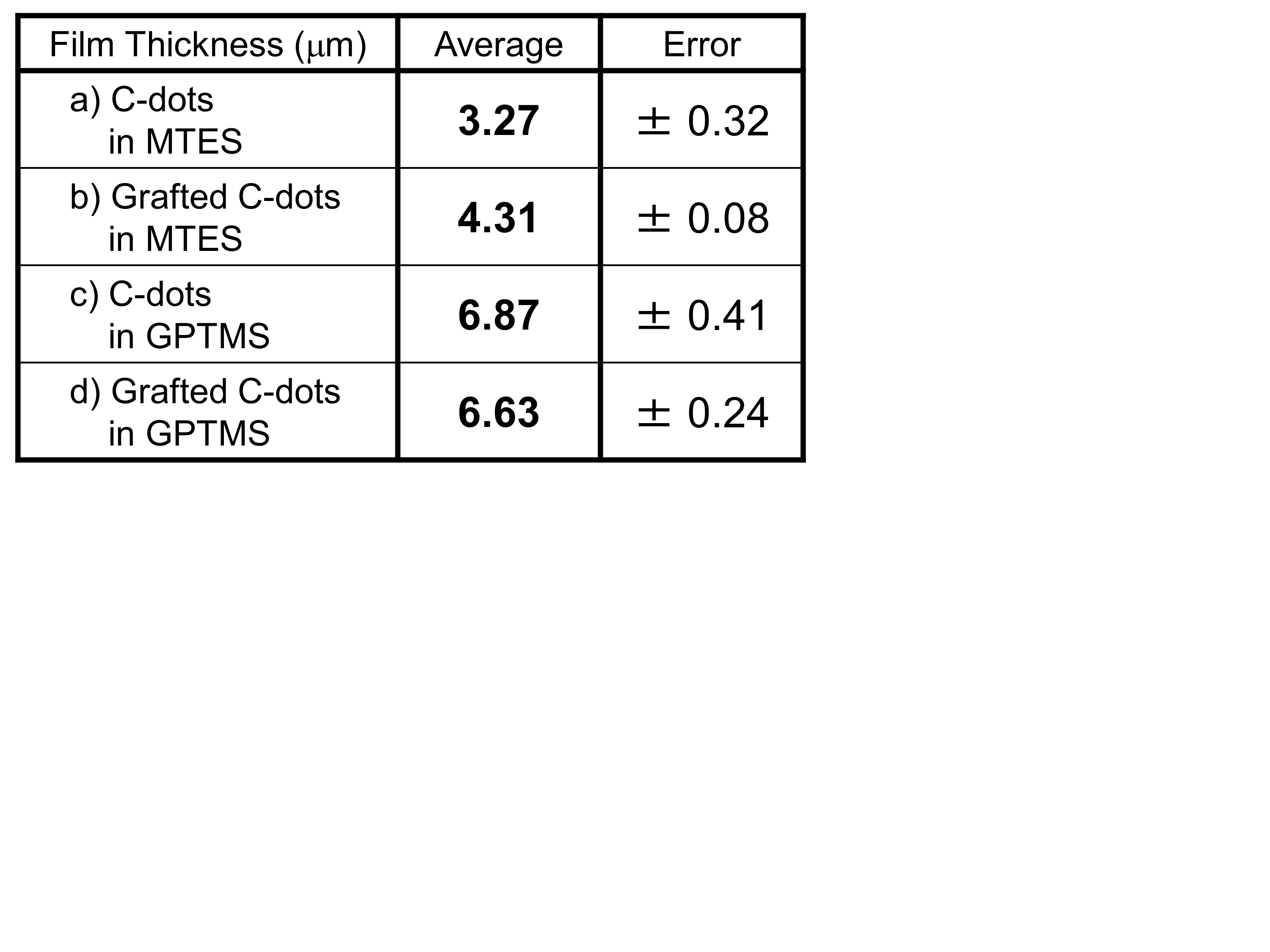

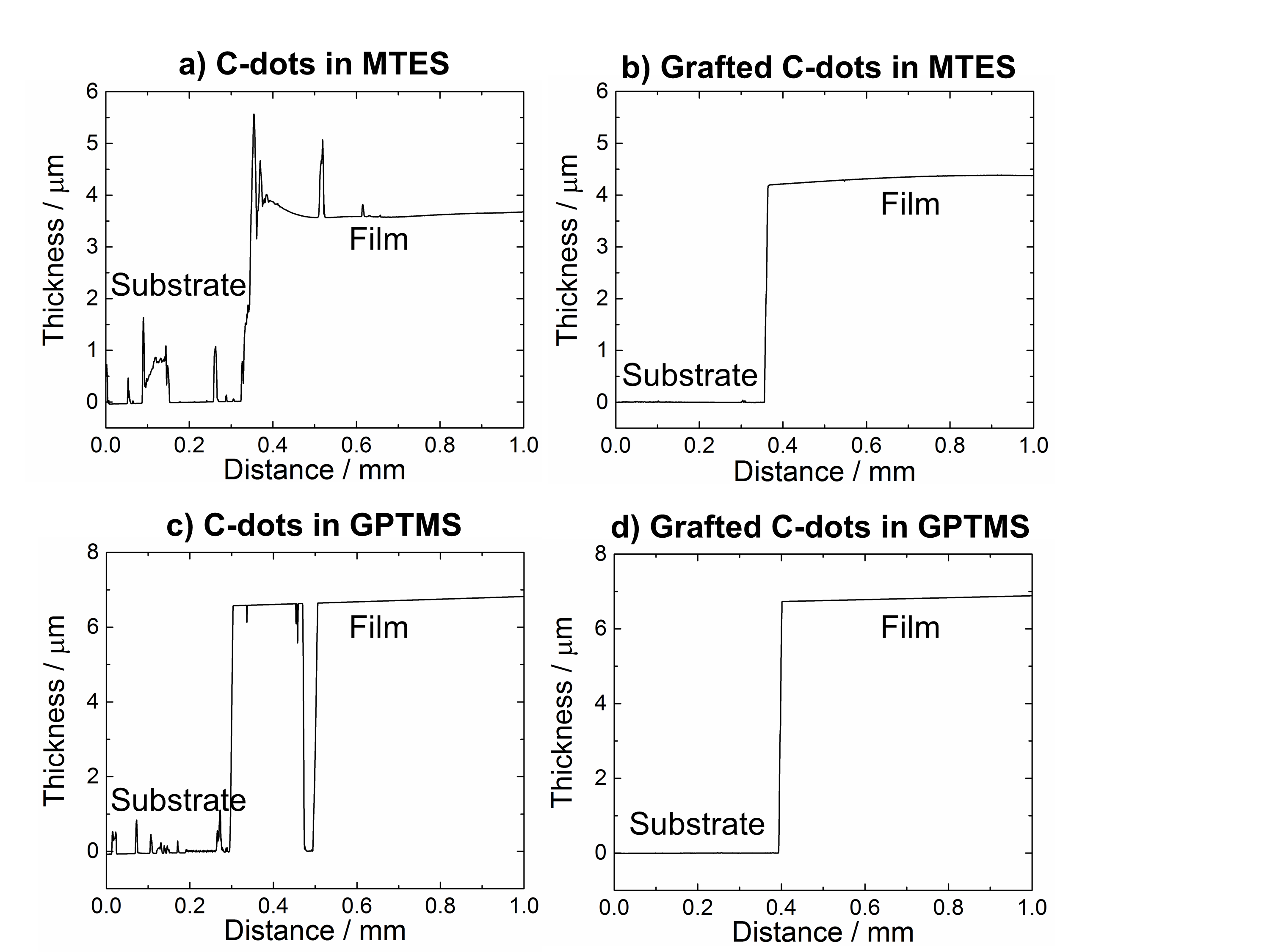


**Figure S3. S**urface morphology and thickness measurements of the films: a) MTES – C-dots, b) MTES – Grafted C-dots, c) GPTMS – C-dots, and d) GPTMS – Grafted C-dots. Table shows the thickness and measurement error for each film. The thickness was determined by the average of the six different points of films. One of measurements for each sample is shown as examples. Film surface is almost flat for all the samples except for the scratch or the residuals. The thickness of the films is almost same regardless of grafting. In the MTES matrix, film thickness is MTES – C-dots: 3.3 μm, and MTES – Grafted C-dots: 4.3 μm, while in the GPTMS matrix, it is GPTMS – C-dots: 6.9 μm, and GPTMS – Grafted C-dots: 6.6 μm.


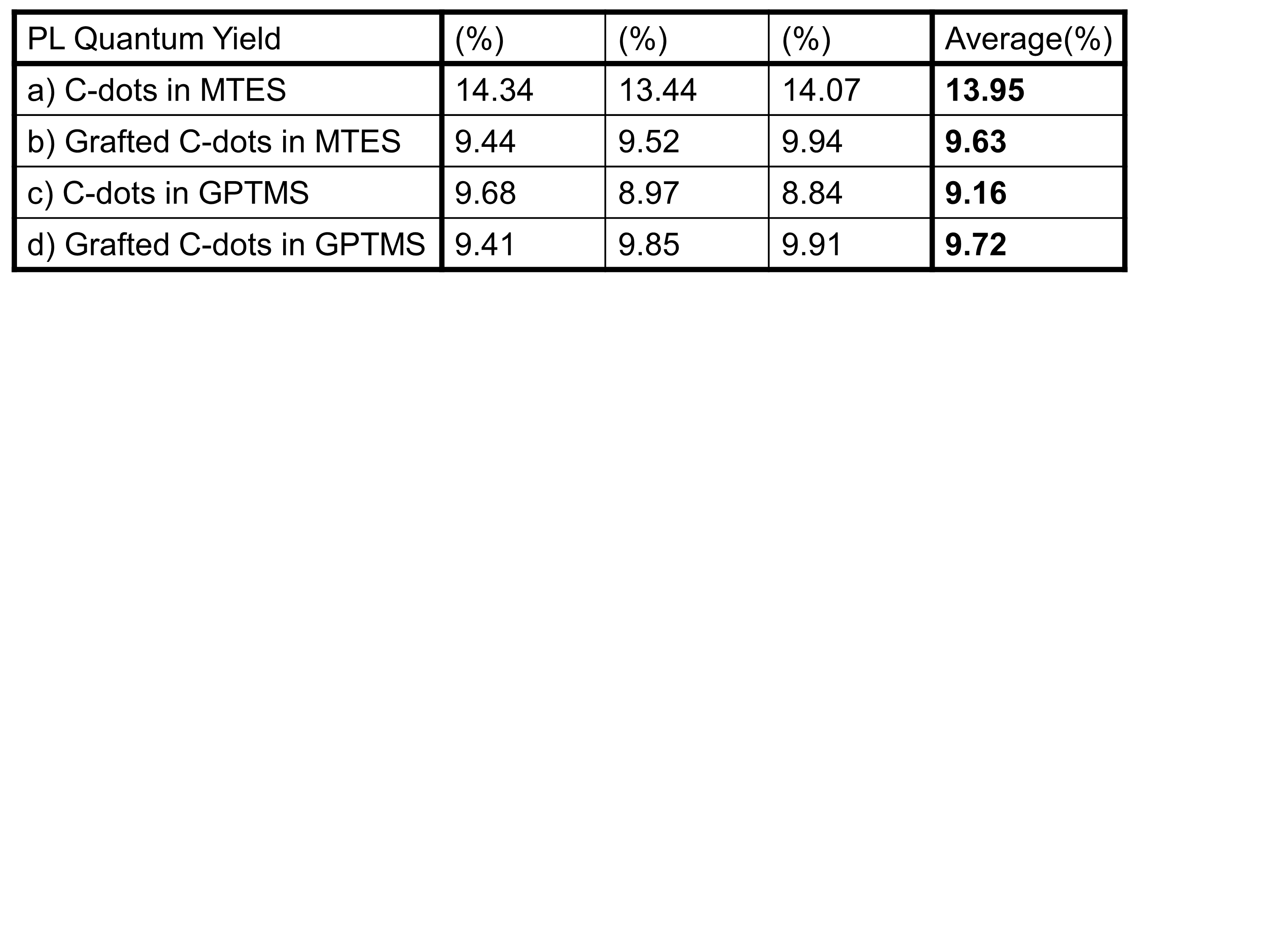

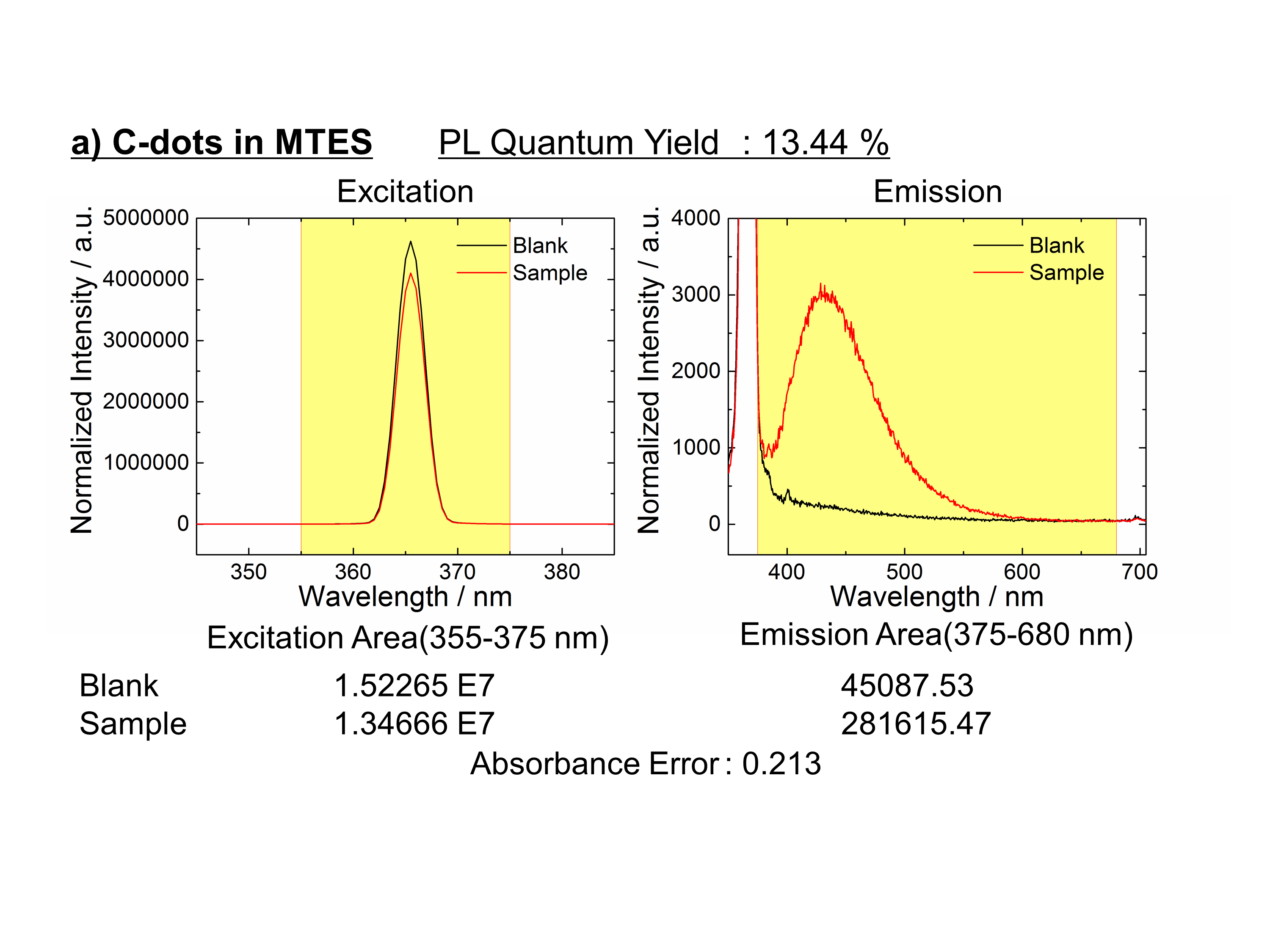

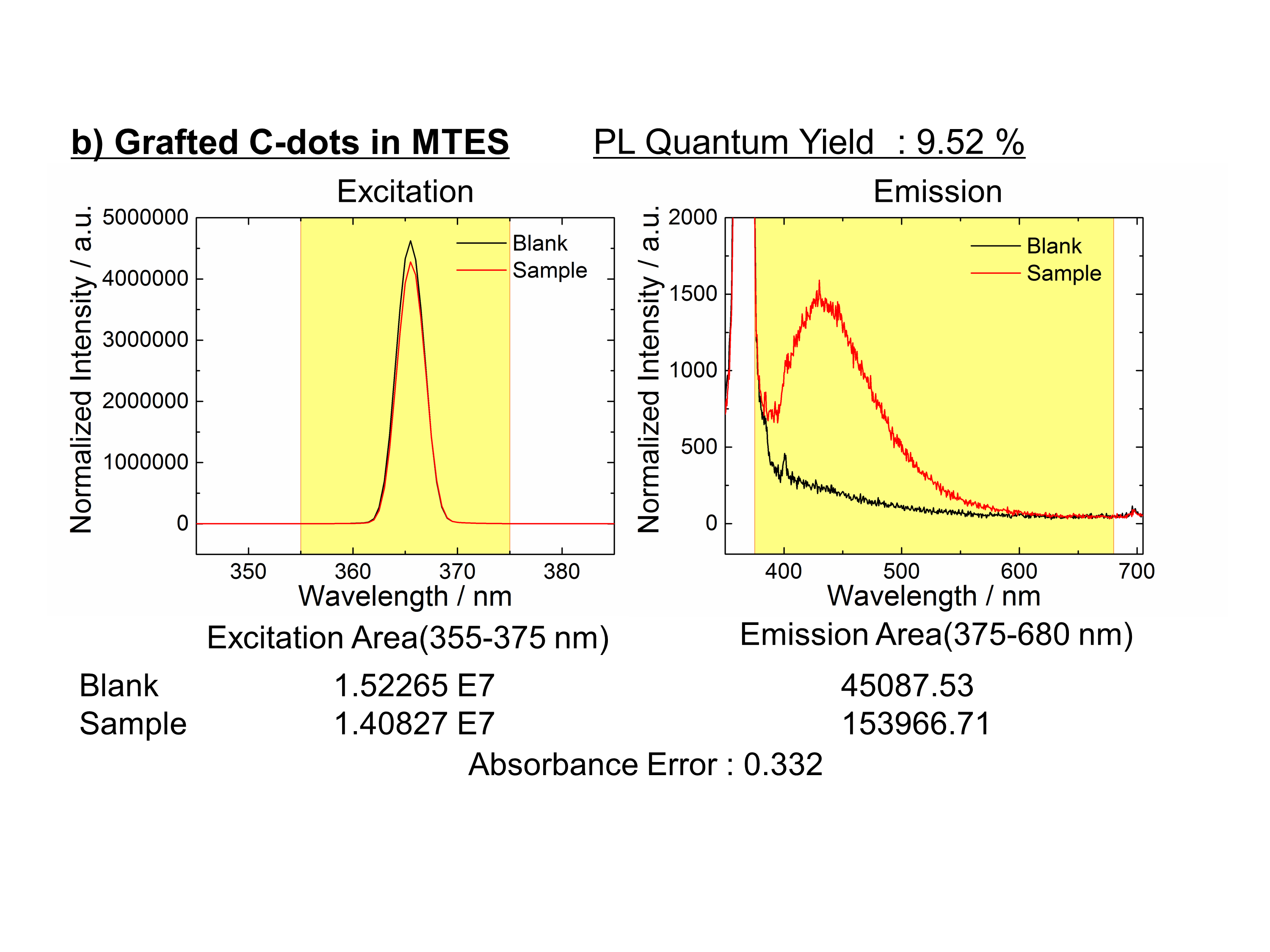

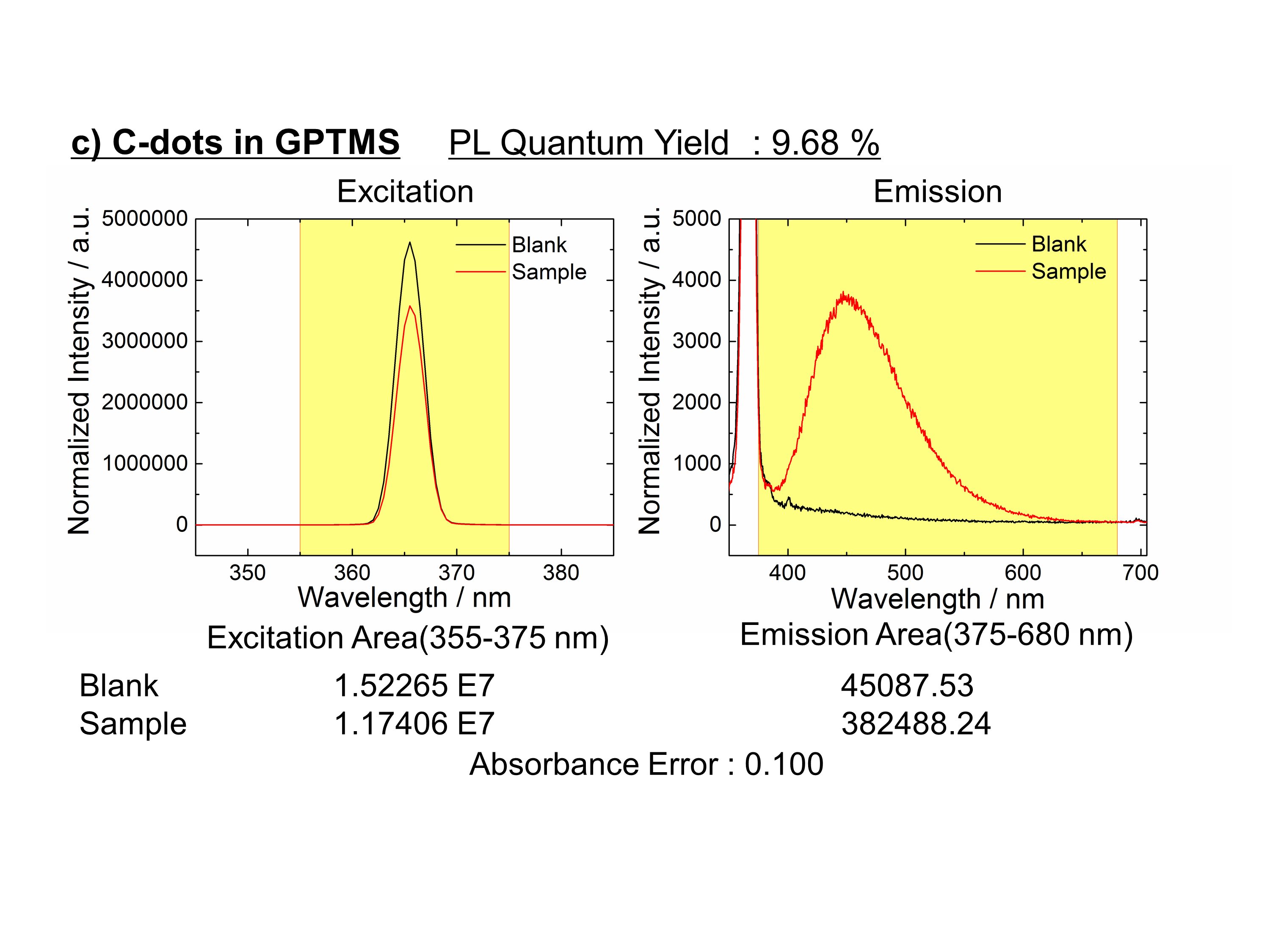

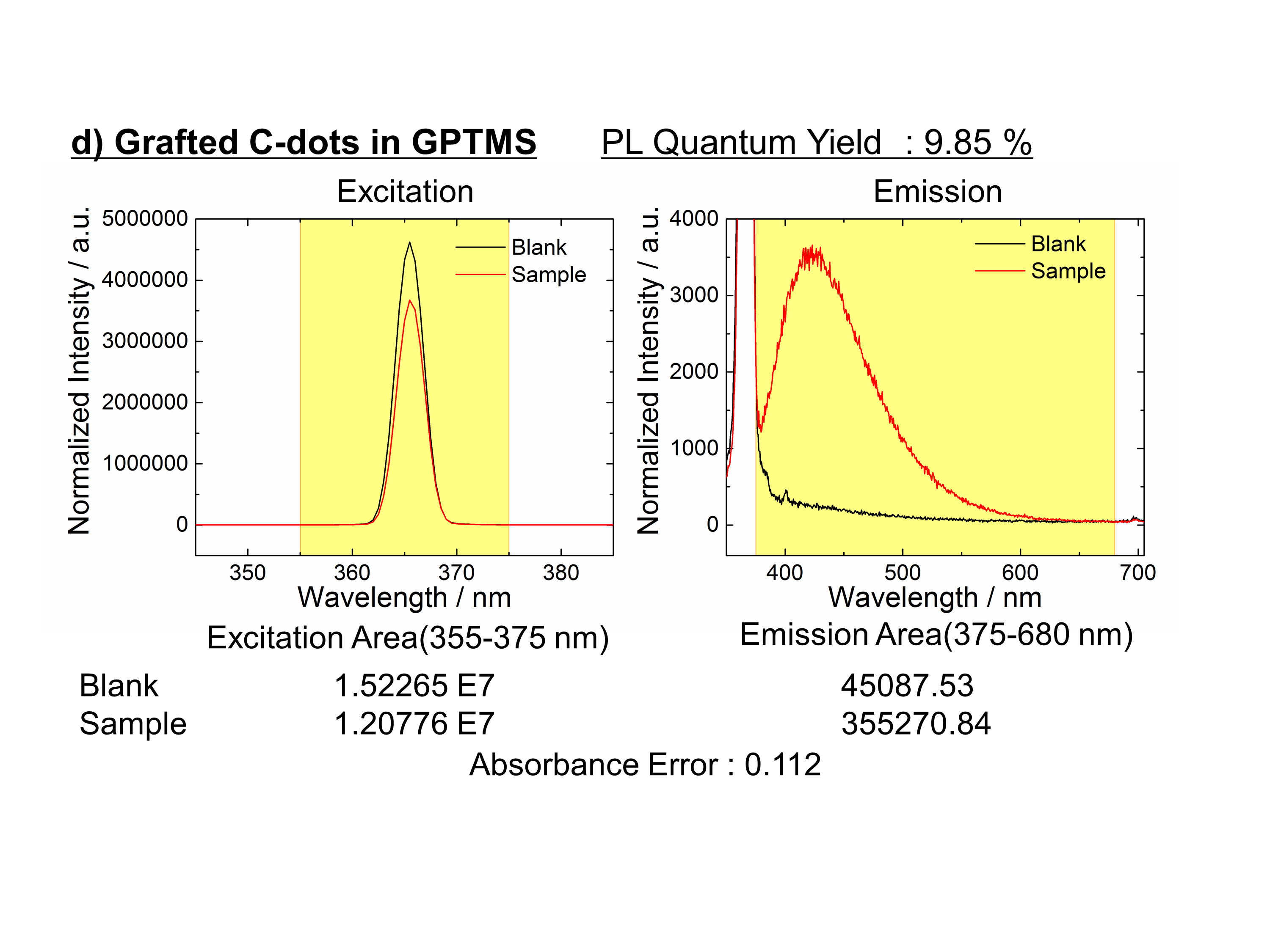


**Figure S4.** Photoluminescence quantum yields (PLQY) of MTES – C-dots, MTES – Grafted C-dots, GPTMS – C-dots and GPTMS – Grafted C-dots films, measured by fluorospectrometer “Nanolog” with an integrated sphere attachment. Table shows the PLQY of each film. Three samples were prepared and measured for each composition, and the PL quantum yields were obtained as an average. One of the examples of the PLQY measurements is shown for each film as a)-d).

The PLQY of each film is more or less the similar yield, around 10%, regardless of the grafting or the matrix. However, MTES – C-dots film shows a little higher value, around 14%.
